# *Elovl4b* knockout zebrafish as a model for ocular very-long-chain PUFA deficiency

**DOI:** 10.1016/j.jlr.2024.100518

**Published:** 2024-02-10

**Authors:** Uzoamaka Nwagbo, Saba Parvez, J. Alan Maschek, Paul S. Bernstein

**Affiliations:** 1 Department of Ophthalmology and Visual Sciences, John A. Moran Eye Center, University of Utah, Salt Lake City, UT, USA; 2 Department of Pharmacology and Toxicology, University of Utah, Salt Lake City, UT, USA; 3 Metabolomics Core, University of Utah Health Sciences Center, Salt Lake City, UT, USA

**Keywords:** zebrafish, ELOVL4, lipids, very-long-chain PUFAs, retinal degeneration

## Abstract

Very-long-chain PUFAs (VLC-PUFAs) are a group of lipids with chain lengths >24 carbons, and the ELOVL4 (elongation of very-long-chain FA-4) enzyme is responsible for vertebrate VLC-PUFA biosynthesis. Studies on the role of VLC-PUFAs in vision have been hindered because of the need for adequate animal models to capture the global loss of VLC-PUFAs. Since homozygous *Elovl4* ablation is lethal in neonatal mice because of catastrophic drying from the loss of their protective skin barrier, we established a zebrafish (*Danio rerio*) model of *Elovl4* ablation. We generated *Elovl4b* KO zebrafish by creating a 56-bp deletion mutation in exon 2 of the *Elovl4b* gene using CRISPR-Cas9. We used GC-MS and LC-MS/MS to analyze the VLC-PUFA and lipid profiles from wild-type and *Elovl4b* KO fish eyes. We also performed histology and visual-behavioral tests. We found that heterozygous and homozygous *Elovl4b* KO zebrafish eyes had altered lipid profiles and a significantly lower C30 to C36 VLC-PUFA abundance than wild-type fish. Moreover, *Elovl4b*^*+/−*^ and *Elovl4b*^*−/−*^ KO larvae had significantly lower motor activity in response to light-dark cycles than their age-matched controls. *Elovl4b*^*−/−*^ adult fish showed no obvious differences in gross retinal morphology and lamination compared with wild type, except for the presence of lipid droplets within the retinal pigment epithelial cell layer of *Elovl4b*^*−/−*^ fish. Our data indicate that the loss of *Elovl4b* in zebrafish changes ocular lipid profiles and leads to visual abnormalities and subtle retinal changes. These findings highlight the use of zebrafish as a model for VLC-PUFA depletion and ELOVL4-related dysfunction.

Very-long-chain PUFAs (VLC-PUFAs) comprise <2% of FAs in the retina and are enriched in photoreceptor outer segment disks. VLC-PUFAs are unique not only because of their chain length but also because they have a saturated FA-like structure near the carboxylic acid proximal group and a distal PUFA tail group, consistent with a bifunctional role in maintaining photoreceptor structural integrity and membrane fluidity (1). They are not present in significant amounts in typical diets. Thus, humans and most animals rely on de novo biosynthesis by ELOVL4 (elongation of very long-chain FA-4) from long-chain PUFA (LC-PUFA) dietary precursors, such as linoleic acid, α-linolenic acid, EPA, DHA, and arachidonic acid (2). VLC-PUFAs are not currently available as dietary supplements, but fish oils enriched in C26 and C28 VLC-PUFAs have been prepared, and we have developed organic synthetic pathways to prepare ≥C30 VLC-PUFAs in gram quantities using organozinc reactions (3). The study of VLC-PUFAs has become prominent because they are implicated in widespread retinal disorders such as diabetic retinopathy and age-related macular degeneration (AMD), for which there currently are no cures (4).

Very-long-chain FAs (VLC-FAs), including VLC-PUFAs, are synthesized by ELOVL4, which is a fatty acyl elongase (or 3-keto acyl-CoA synthase) expressed in the retina, testes, skin, and brain that is responsible for the initial rate-limiting step and subsequent elongation steps in the biosynthesis of VLC-FAs (5). Patients with Stargardt 3 disease (STGD3), an early-onset macular dystrophy, have C-terminal *ELOVL4* mutations that lead to a premature stop in ELOVL4 protein translation, creating a truncated transmembrane protein without an endoplasmic reticulum dilysine retention signal (KXKXX). The three identified mutations associated with autosomal dominant STDG3 occur in exon 6 of *ELOVL4* (2). These mutations occur downstream of the dideoxy iron-binding motif (HXXHH), which is in the catalytic domain of the elongase. Although these mutant forms of ELOVL4 are reported not to be catalytically active, cell culture studies have shown that mutant proteins also mislocalize and aggregate in the cytoplasm, causing cellular stress and photoreceptor cell death (5, 6). The combined effects of protein mislocalization and reduced levels of retinal VLC-PUFAs because of haploinsufficiency are thought to underlie the retinal pathology seen in patients with STGD3. In addition, age-related declines in ELOVL4 and ELOVL2 function may contribute to low levels of retinal VLC-PUFAs associated with AMD (2, 7).

The retinal effects of homozygous ELOVL4 KO mutations in humans are poorly characterized because they usually result in early death. Less severe recessive mutations in ELOVL4 in humans are typically toward the N-terminus of the protein. These N-terminal mutations have been associated with nervous system disorders (spinocerebellar ataxia), skin disorders (ichthyosis), and reproductive disorders (8, 9, 10). Recent work indicates that these mutations predominantly affect the enzyme’s ability to make very-long-chain saturated FAs (VLC-SFAs) rather than VLC-PUFAs; so, unsurprisingly, these individuals have not been reported to have a retinal phenotype.

Various mouse models have been developed to study STDG3 mutations in vivo. Still, the complete ablation of *Elovl4* is lethal in neonatal mice because of the loss of VLC-SFA-containing ceramides in the skin, which leads to subsequent dehydration (11). *Elovl4* KO mice whose skin phenotype was rescued by the expression of an *Elovl4* transgene in their skin suffered from severe seizures and died in the first month of life before retinal degeneration phenotypes became apparent (9). In addition, a separate study showed a dose-dependent relationship in the observed severity of disease phenotypes because of the overexpression of the STGD3-specific Elovl4 mutation in mouse photoreceptors (12). So far, the conditional knock-in of human *STGD3* mutations and the conditional KO of *Elovl4* in mouse ocular tissue present with varying phenotypes and take a relatively long time to deplete VLC-PUFAs and manifest disease progression (13, 14). Thus, with the current rodent models, it is still difficult to fully recapitulate the human disease phenotype, and it is unclear if VLC-PUFA depletion alone is sufficient to cause significant retinal pathology.

Zebrafish (*Danio rerio*) have gained popularity as tools for lipid metabolism and retinal degeneration research as they are genetically similar to mammals, robust, and have quick generation times. They are a class of bony fish (teleosts) that live in freshwater in the tropical subregion of India. They have retinal structures, cell types, and cone-rich retinas reminiscent of the cone-dense macula in higher-order primates. Moreover, many genes relevant to human disease research are evolutionarily conserved and duplicated in the zebrafish genome. Interestingly, zebrafish gene homologs for many members of the ELOVL elongase family have been identified (10, 15). Elovl4a, which catalyzes the formation of VLC-SFAs, is expressed in most zebrafish tissues, including the brain, other neuronal tissue, skin, and liver. Meanwhile, Elovl4b, which primarily catalyzes the production of VLC-PUFAs, is restricted to the gonads, pineal gland, and retinal photoreceptor layer (10). In addition, the VLC-PUFA biosynthetic pathway is relatively conserved between fish and humans (Fig. 1). As in mammals, VLC-PUFAs greater than 26 carbons in chain length are thought to be exclusively synthesized by Elovl4b on a similar biosynthetic pathway consisting of other elongases, β-oxidases, and FA desaturases. Moreover, their retinal cell types and structure are conserved and comparable to humans. Although zebrafish, like rodents, do not possess maculas, their cone-rich retinas provide a valuable tool for understanding human pathologies.

**Fig. 1:**
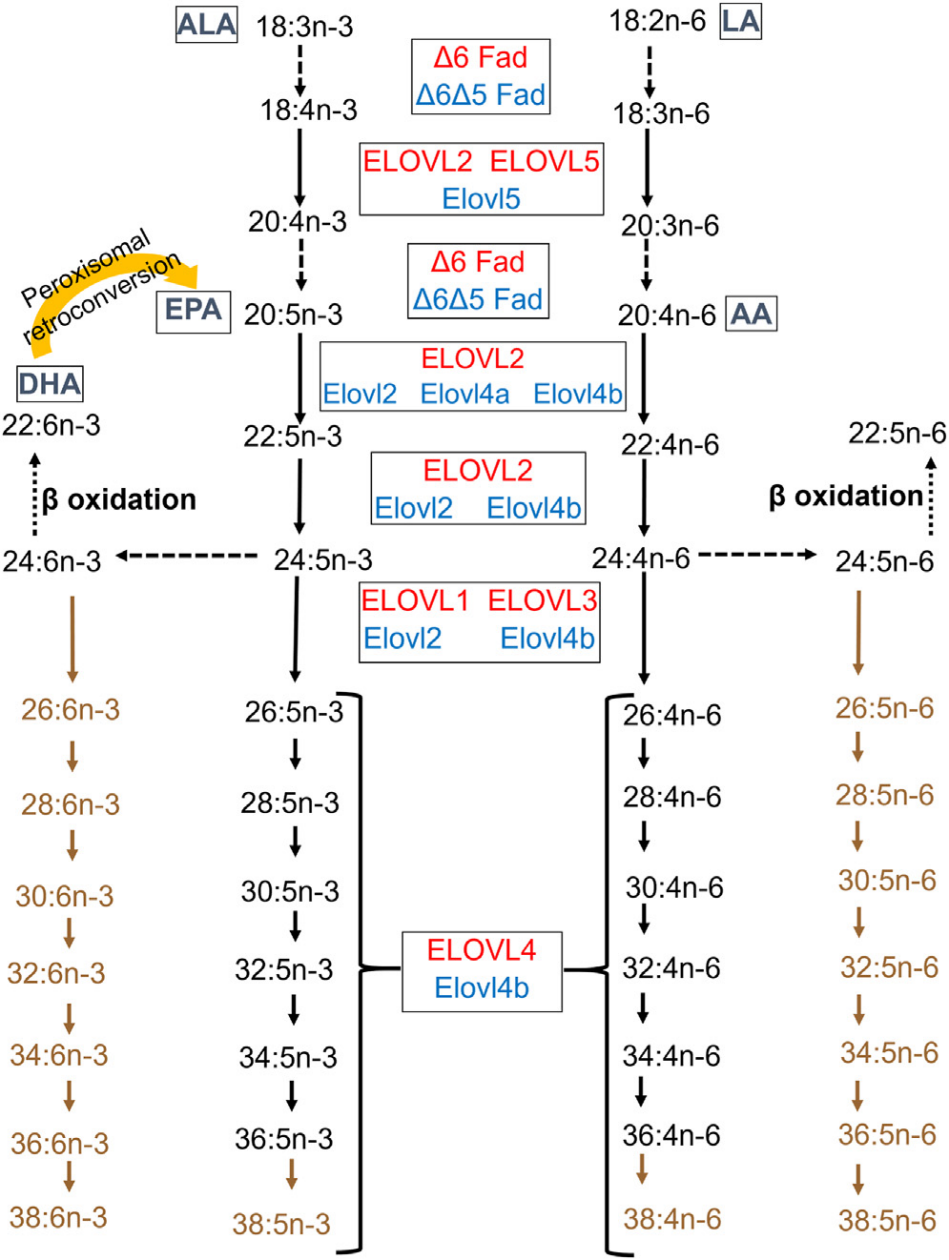
VLC-PUFA biosynthetic pathways in mammals and zebrafish. Comparison of the VLC-PUFA biosynthetic pathways from linoleic (18:2n-6) and α-linolenic (18:3n-3) acid precursors of zebrafish and mammalian vertebrates (adapted from Refs. (10) and (13)). Large dashed black arrows represent the desaturation steps, small dashed arrows represent the β-oxidation steps, and solid arrows represent the elongation steps. AA, arachidonic acid; ALA, α-linolenic acid; LA, linoleic acid. Color key: red text: mammalian (human and murine) orthologues, blue text: zebrafish orthologues, and brown text and arrows: pathway presumed but not confirmed in zebrafish.

Until now, the effects of complete or partial loss of VLC-PUFAs on visual behavior and retinal morphology were unclear in the absence of confounding effects of protein mislocalization and aggregation. This study investigates the impact of homozygous and heterozygous germline deletion mutations of *Elovl4b* on ocular lipids, VLC-PUFAs, visual function, and ocular morphology in adult and larval zebrafish.

## Materials and methods

### Zebrafish husbandry

AB strain zebrafish (Zebrafish International Research Center, Eugene, OR) were used as wild-type experimental controls and to generate deletion mutants. Adult zebrafish were maintained by the University of Utah’s Centralized Zebrafish Core Facility under a controlled 14 h light/10 h dark cycle. They were fed a commercial diet of 3–5% body weight per day of Zeigler® zebrafish granules and SPAROS® zebrafish feed containing essential amino acids, vitamins, minerals, FAs, and marine phospholipids twice daily in a recirculating aquarium system. The Institutional Animal Care and Use Committee of the University of Utah approved the breeding and experimental procedures used.

### Zebrafish *Elovl4b* ablation and stable line generation

To prepare for CRISPR-Cas9 ribonucleoprotein complex microinjection into zebrafish embryos, CHOPCHOP (https://chopchop.cbu.uib.no) was used to determine guide RNA sequences targeting zebrafish *Elovl4b* with minimal predicted off-target effects (16). Two *Elovl4b*-targeting guide RNA sequences in exon 2 were selected, 5′ TCTCCTTACCTGCTATGGTAAGG 3′ and 5′ CAGGTGAACGACCGTCTCCATGG 3′. Single guide RNA (sgRNA) oligonucleotides and Cas9 nuclease (Alt-R® S.p. Cas9 Nuclease V3, 100 μg [catalog no.: 1081058]) were purchased from Integrated DNA Technologies, Coralville, IA. The CRISPR-Cas9 ribonucleoprotein complex was assembled by mixing 2.5 μM of each sgRNA complex solution with 5 μM Cas9 protein solution diluted in Cas9 working buffer (20 mM Hepes-NaOH [pH 7.5], 350 mM KCl, and 20.5% glycerol), and nuclease-free water, after which the mixture was incubated at 37°C for 5 min and returned to room temperature shortly before microinjection into one-cell stage zebrafish embryos.

### Genotyping PCR

Genomic DNA was extracted from caudal fin clips of adult zebrafish. Fin tissue was incubated in 75 μl lysis buffer (40 mM NaOH, 0.2 mM EDTA) for 1 h at 98°C. The lysed samples were diluted with 75 μl of 40 mM Tris-HCl (pH 5.5) and centrifuged for 1 min. Then 2 μl of the supernatant was incubated with 0.5 μM forward (5′ CTGCTTCTGTTGTGTTTTCTGC 3′) and reverse (5′ ACACAAATGCTTGGCACAATTA 3′) primers, 7 μl ddH_2_0, and 10 μl 2× M-PCR OPTI™ Mix (Dye Plus) (Bimake; catalog no.: B45012) in a total volume of 20 μl. Samples were denatured at 94°C for 5 min, followed by 28 cycles of amplification consisting of 30 s at 94°C, 30 s at 60.1°C, and 30 s at 72°C, followed by a final primer extension of 7 min at 72°C. Deletion mutations were identified by gel electrophoresis.

### Establishment of an *Elovl4b*^*−/−*^ zebrafish line

F_0_ mosaic fish were outcrossed with wild-type AB fish to confirm germline transmission of the deletion mutation and to screen out and minimize nonspecific off-target mutations. Heterozygous mutants were mated to generate homozygous *Elovl4b*^*−/−*^ mutants that were crossed for subsequent generations. This stable line was established following protocols outlined by Moravec and Pelegri (17). Genomic DNA from *Elovl4b*^*−/−*^ fish was extracted and purified by gel electrophoresis to sequence the deletion mutation. DNA extraction was performed using the DNeasy® Blood & Tissue Kit from Qiagen, Inc. For PCR, samples were denatured at 94°C for 5 min, followed by 30 cycles of amplification consisting of 2 min at 94°C, 30 s at 62.4°C, and 30 s at 72°C, followed by a final primer extension of 7 min at 72°C. The primers used to amplify the region for sequencing were forward 5′ TGTCATTGGGAGATGAGCAA 3′ and reverse 5′ GTAAAGGCCCATCTCACTGG 3′. PCR was performed to amplify the sequence around the region of interest with primers spanning 627 bp around the target site for the deletion mutation. PCR products were purified by gel electrophoresis, extracted with QIAquick® Gel Extraction Kit, suspended in 30 μl Buffer EB, and sequenced by the University of Utah’s Sequencing Core facility.

### RT-PCR

Fish were euthanized by ice-chilled water immersion. After death, brain and retinal tissue were isolated through microdissection and homogenized using Fisherbrand™ RNase-free disposable pellet pestles. They were stored in liquid nitrogen until RNA extraction. Total RNA from brains and retinal tissue of age- and sex-matched wild-type, heterozygous, and homozygous *Elovl4b* KO fish was isolated using a Qiagen RNeasy mini kit following the manufacturer’s protocol. RNA was quantified using a NanoDrop spectrophotometer (Thermo Scientific). For the determination of mRNA expression levels and to validate germline mutagenesis, total RNA was reverse transcribed with a Verso cDNA Synthesis Kit (ThermoFisher) according to the manufacturer’s protocols using gene-targeting primers synthesized by the University of Utah DNA Peptide Synthesis core facility. The primer sets used are detailed in supplemental Table S1. The resultant RT-PCR products were resolved by gel electrophoresis on 2.5% agarose and imaged using an Invitrogen iBright™ CL750 Imager (Carlsbad, CA).

### Retinal histology

To assess gross retinal morphology, 1-year-old zebrafish heads were fixed in 10% neutral-buffered formalin and shipped to HistoWiz (https://home.histowiz.com) for gross processing, paraffin wax embedding, sectioning, and staining with toluidine blue. Pathogenic subretinal pigment epithelium (RPE) lipid deposition is a characteristic of retinal degenerative diseases like AMD (18, 19). We assessed changes in retinal morphology and lipid deposition of retinal sections stained with oil red O (ORO). Whole eyes were isolated from 13-month-old male zebrafish euthanized by ice water immersion. Right eyes were fixed in 4% paraformaldehyde overnight and then incubated in 15% sucrose and 30% sucrose overnight at 4^o^C. The lenses were removed, and the eye cups oriented in cryomolds with Tissue-Tek® optimum cutting temperature compound, Sakura® Finetek (VWR; catalog no.: 25608-930), with the nasal sides of the eye cups directed into the mold. The eye cups in optimum cutting temperature compound were then flash frozen with isopentane before storage at −80^o^C prior to sectioning. A Leica cryostat was used to make ∼10–12 μm thick sections, which were placed on SuperFrost Plus microscope slides (catalog no.: 12-550-15; Fisher Scientific, Pittsburgh, PA). Sections were stained according to protocols used by Noel *et al.* (20). Briefly, sections were brought to room temperature before being stained with 0.3% ORO in 60% isopropanol solution, rinsed with 60% isopropanol, and then counterstained with hematoxylin. Slides were mounted in Fluoromount-G® mounting medium (catalog no.: 0100-01; SouthernBiotech, Birmingham, AL) before imaging at 40× with a Zeiss AxioScan slide scanner.

### Visual motor response assay and behavioral tests

A detailed description of the visual motor response (VMR) assay and behavioral tests can be found in the Supplemental data.

### GC-MS and LC-MS/MS analyses

Omega-3 (n-3) and omega-6 (n-6) VLC-PUFAs were analyzed using GC-MS and previously published protocols (2). For total lipid analyses, we adapted the protocol used by Harkiewicz *et al.* (21) for lipid extraction from zebrafish tissue and LC-MS/MS. A detailed description of the sample preparation and MS conditions of VLC-PUFA and lipidomics analyses can be found in the Supplemental materials and methods.

## Results

### *Elovl4b* KO zebrafish are fertile and produce viable offspring

**Fig. 2:**
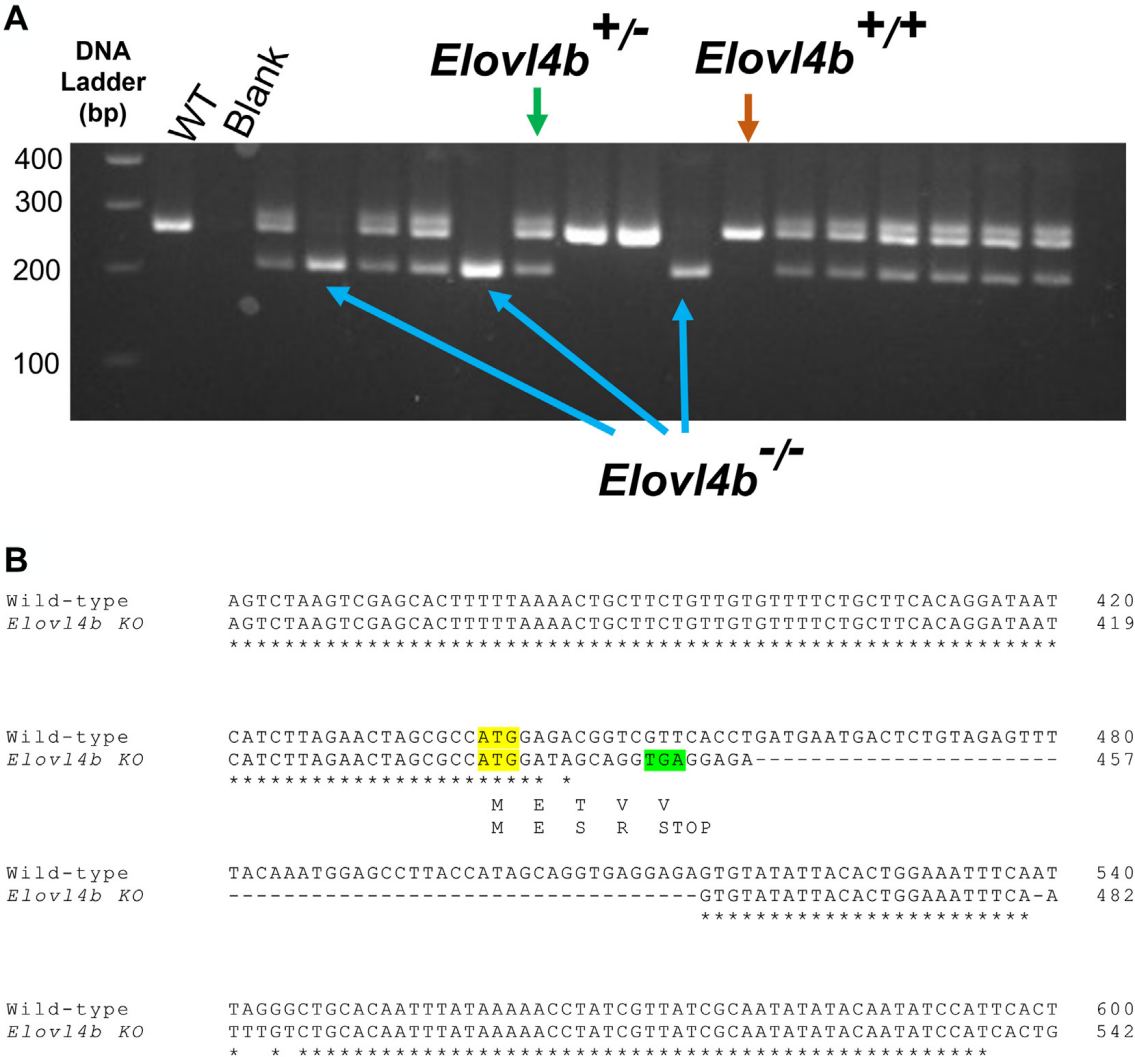
Sequencing and PCR analysis. A: Gel electrophoresis showing wild-type, heterozygous, and homozygous *Elovl4b* KO zebrafish. DNA was extracted from caudal fin clips and amplified using PCR. The PCR product was subsequently run on 2% agarose gel at 110 V. This image was captured using an iBright CL750 imaging system (Invitrogen, Carlsbad, CA). B: Nucleotide sequence alignment between wild-type and homozygous *Elovl4b* mutant fish shows that a 56 bp deletion mutation at exon 2 of zebrafish *Elolv4b* leads to a premature termination codon at position 5 of the protein-coding sequence. The start codons are highlighted in yellow, and the premature termination codon is highlighted in green.

**Fig. 3:**
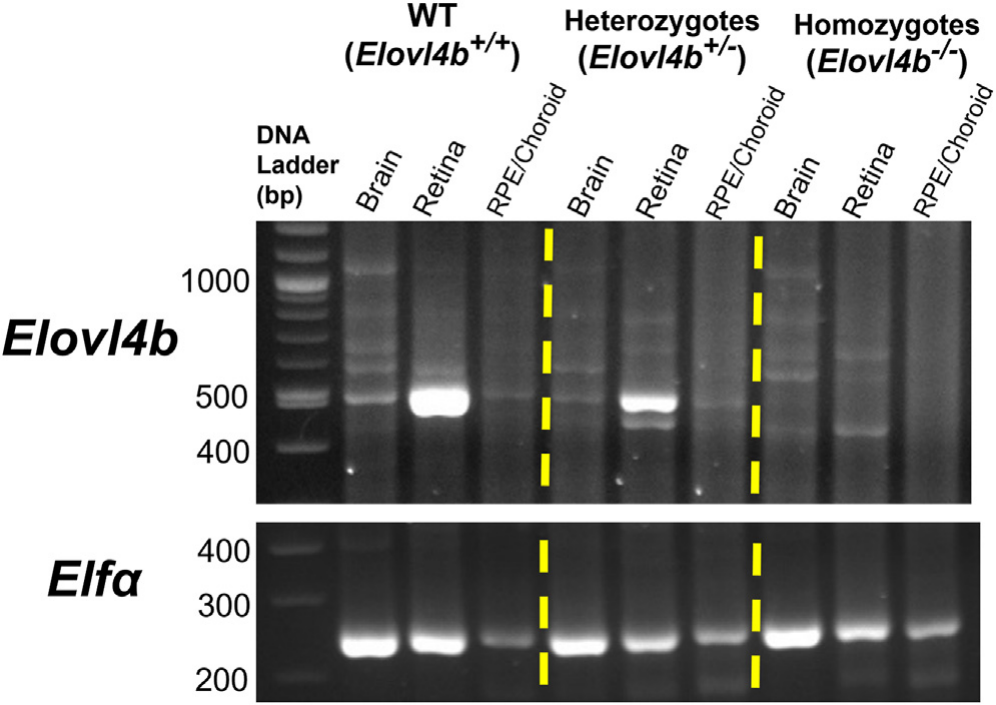
RT-PCR analysis of brain and ocular tissue from *Elovl4b* KO fish. Gel electrophoresis of RT-PCR products at their expected molecular weight. *Elovl4b* expression is reduced in brain and retina lysates in heterozygous and homozygous *Elovl4b KO* fish compared with WT controls.

ELOVL4 is responsible for the rate-limiting step in vertebrate VLC-PUFA biosynthesis. Zebrafish Elovl4a and Elovl4b are conserved between zebrafish and their mammalian orthologs. A discussion of the conserved motifs can be found in the Supplemental data and supplemental Fig. S1. We targeted exon 2 of *Elovl4b* to test the effect of VLC-PUFA depletion in the absence of protein formation because exon 2 is far upstream of the STGD3 mutations on exon 6 of human *ELOVL4*—which cause C-terminal truncation and mislocalization of ELOVL4. The deletion mutation on exon 2 was near to and downstream of the start codon to improve the chances of a missense mutation, which would minimize the possibility of functional protein formation (22). To generate *Elovl4b* KO fish, we injected Cas9 nuclease duplexed with two sgRNA oligonucleotides targeting exon 2 of *Elovl4b* to create a 56-bp deletion mutation in *Elovl4b* for simpler screening via PCR and gel electrophoresis. Surviving embryos were raised to adulthood and screened for germline mutations by outcrossing with wild-type AB strain zebrafish. The F_1_ generation of *Elovl4b* KO fish with our desired germline mutation was then crossed to produce F_2_ fish with wild-type, heterozygous, and homozygous *Elovl4b* deletion mutations (Fig. 2A). To confirm *Elovl4b* deletion, we sequenced the *Elovl4b* KO mutant and identified a 56-base pair deletion mutation and premature termination codon at amino acid position 5 of Elovl4b′s 303 amino acid residues (Fig. 2B). This deletion generates a fish with the mutation: *Elovl4b* c.187_242del p.T3SfsX3. We also performed RT-PCR analyses and found that the signal for *Elovl4b* gene expression was reduced in the brain and retinas of heterozygous *Elovl4b* mutant fish and undetectable in homozygous *Elovl4b* mutants (Fig. 3). Although ELOVL4 is expressed in the testes of mammalian vertebrates, *Elovl4b* ablation did not affect the fertility of the fish, as male and female *Elovl4b*^*−/−*^ zebrafish appeared normal and produced clutch sizes comparable to wild type when mated.

### *Elovl4b* ablation leads to the loss of C30-C36 VLC-PUFAs and changes in major lipid groups

**Fig. 4:**
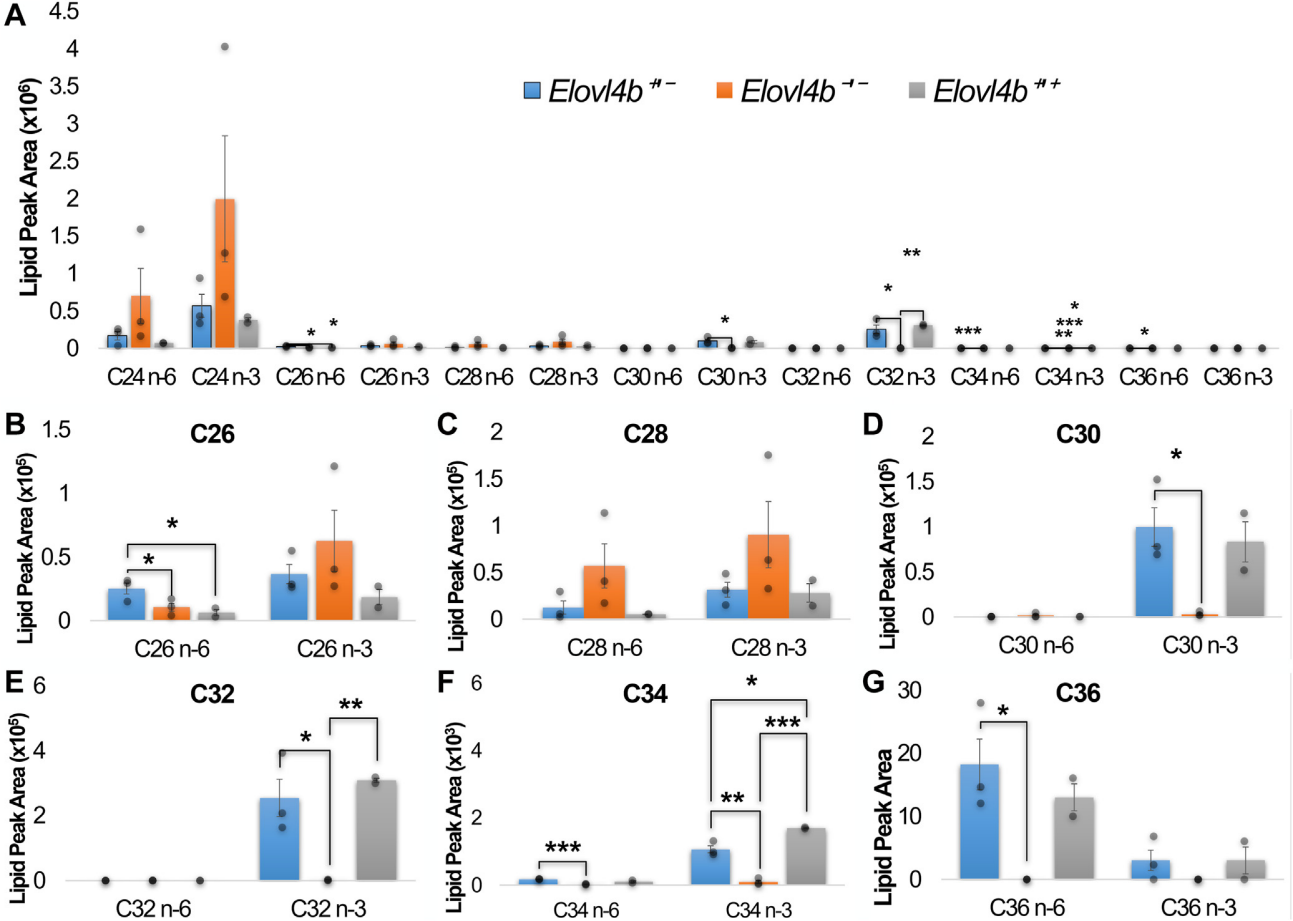
Lipid profile of C24-C36 N-3 and N-6 VLC-PUFAs in *Elovl4b*^*+/−*^ (blue), *Elovl4b*^*−/−*^ (orange), and wild-type *Elovl4b*^*+/+*^ (gray) zebrafish eyes. A: C24-C36, (B) C26, (C) C28, (D) C30, (E) C32, (F) C34, and (G) C36 n-3 and n-6 PUFAs. VLC-PUFA analysis was done using gas chromatography and mass spectrometry. *Elovl4b*^*+/−*^*n* = 3, *Elovl4b*^*−/−*^*n* = 3, and *Elovl4b*^*+/+*^*n* = 2. Lipid peak areas were normalized to the internal standard. Error bars represent the standard error of the mean. *P* values between groups were obtained using a one-tailed *t*-test with unequal variance. ∗*P* ≤ 0.05, ∗∗*P* ≤ 0.01, and ∗∗∗*P* ≤ 0.001.

Mammalian ELOVL4 is known to be responsible for the synthesis of VLC-PUFAs >24 carbons in chain length. To investigate the effect of *Elovl4b* ablation on ocular VLC-PUFAs, we compared the levels of ocular C24–C36 VLC-PUFAs in wild-type and homozygous *Elovl4b* KO fish eyes. We found via GC-MS analysis that the deletion of *Elovl4b* increased the levels of C24 to C28 VLC-PUFAs relative to wild type and diminished the levels of C30 to C-36 n-3 and n-6 VLC-PUFAs in their eyes to nearly undetectable levels. Moreover, fish heterozygous for our *Elovl4b* mutation showed variable intermediate levels of VLC-PUFAs (Fig. 4A–G).

**Fig. 5:**
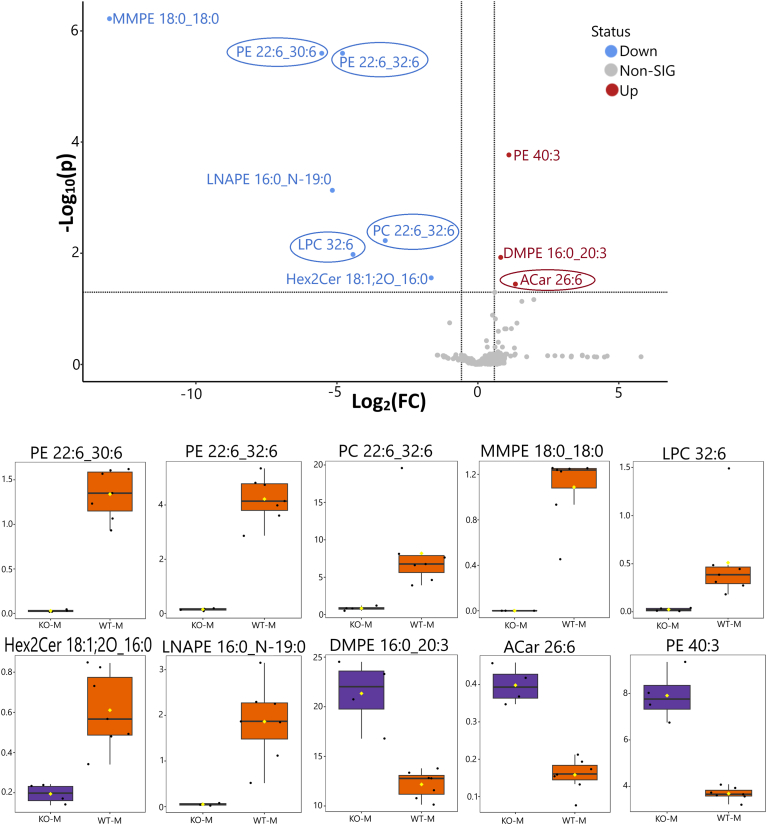
Volcano and box plot analyses of ocular lipid profiles of male *Elovl4b KO* and WT zebrafish normalized to tissue weight using a one-way ANOVA test. Volcano plot shows that seven compounds were significantly downregulated, whereas three compounds were significantly upregulated in ocular lipid comparisons between homozygous *Elovl4b* KO fish and WT age- and sex-matched controls (*n* ≥ 4 each). Blue dots represent significantly downregulated lipid species; red dots represent significantly upregulated lipid species; and gray dots represent lipid groups that were not significant in the comparison using our parameters. VLC-PUFA-containing species are circled in volcano plot comparisons. The box plots show the relative peak intensities of significantly altered lipids. Male WT (WT-M; orange) and homozygous *Elovl4b KO* fish (KO-M; dark purple) (*n* ≥ 4 each). The *y*-axes represent the relative peak intensities obtained through LC-MS/MS analysis. Significance was determined using the following parameters: *P* < 0.05, fold change >1.5, and false discovery rate adjusted.

**Fig. 6:**
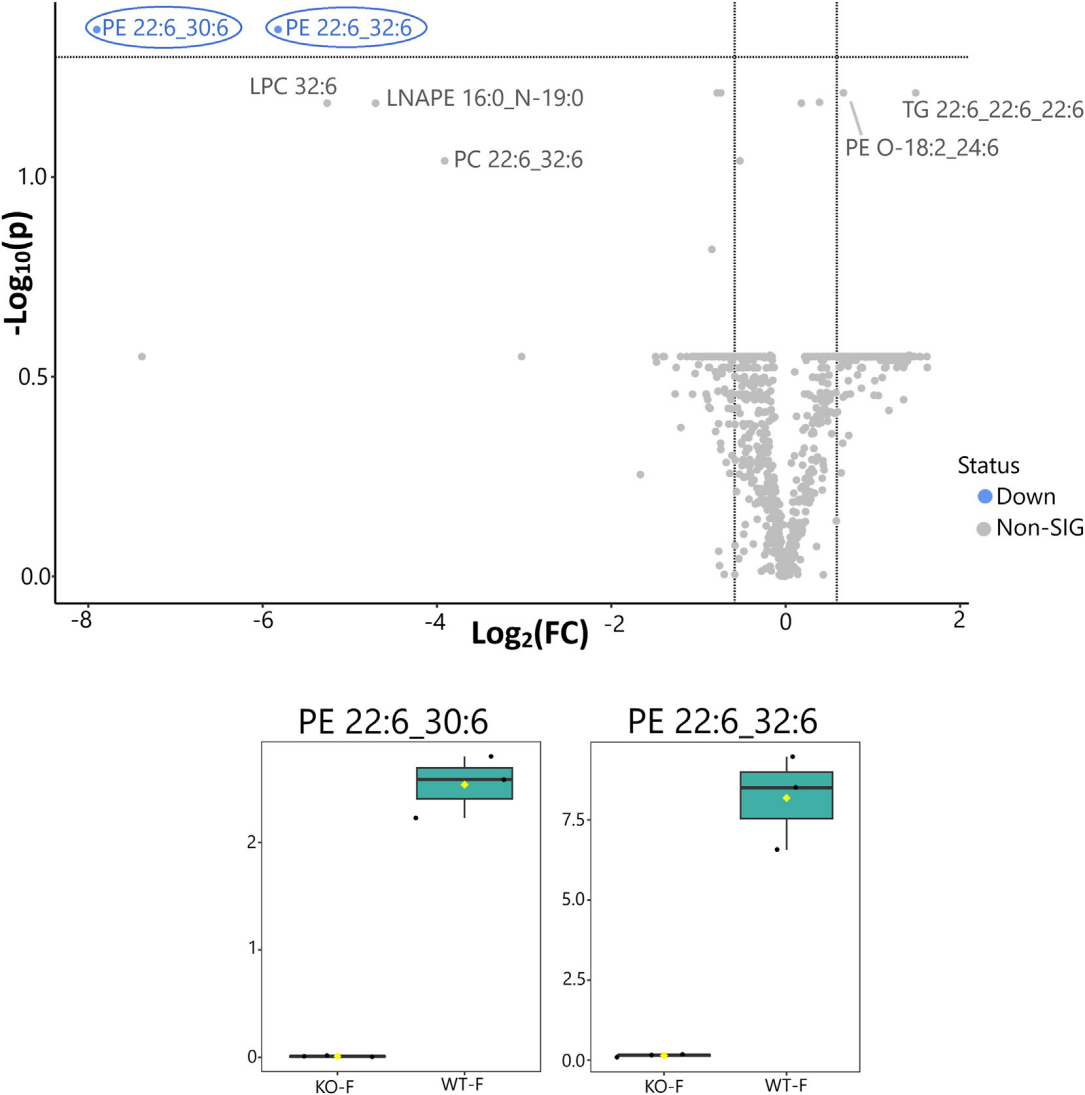
Volcano and box plot analyses of ocular lipid profiles of female *Elovl4b KO* and WT zebrafish normalized to tissue weight using a one-way ANOVA test. Volcano plot analysis showing two compounds significantly downregulated in female homozygous *Elovl4b* KO fish compared with WT age- and sex-matched controls (*n* = 3 each). Blue dots represent significantly downregulated lipid species; and gray dots represent lipid groups that were not significant in the comparison using our parameters. VLC-PUFA-containing species are circled in volcano plot comparisons. The box plots show the relative peak intensities of significantly altered lipids. Female WT (WT-F; teal green) and homozygous *Elovl4b KO* fish (KO-F; tan brown) (*n* = 3 each). The *y*-axes represent the relative peak intensities obtained through LC-MS/MS analysis. Significance was determined using the following parameters: *P* < 0.05, fold change >1.5, and false discovery rate adjusted.

An untargeted lipidomic analysis also revealed significant decreases in the relative abundance of major lipid classes between wild-type control and homozygous *Elovl4b* KO fish. We evaluated the lipid profiles by genotype and sex. We found 10 compounds to be significantly different between the male wild-type and homozygous *Elovl4b* mutant fish (Fig. 5), whereas only two of these compounds were significantly different between these genotypes among the female fish (*P* < 0.05; fold change >1.5; false discovery rate, adjusted) (Fig. 6).

C30 and C32 VLC-PUFA-containing phosphatidylethanolamines (PE 22:6_30:6 and PE 22:6_32:6) were significantly reduced in female and male *Elovl4b*^*−/−*^ fish compared with their wild-type counterparts. Moreover, male *Elovl4b*^*−/−*^ fish had reduced levels of C32 VLC-PUFA-containing PCs (PC 22:6_32:6 and lysophosphatidylcholine 32:6), in addition to other lipid groups like monomethyl-phosphatidylethanolamine 18:0_18:0 and lyso-N-acyl-phosphatidylethanolamine (LNAPE 16:0_N-19:0), which have not previously been associated with ELOVL4 activity. LNAPEs are low-abundance intermediates in the biosynthesis of *N*-acylethanolamines (NAEs). NAEs are lipid mediators involved in regulating homeostatic processes, including energy balance, metabolism, nociception, and inflammation (23). Low levels of LNAPEs (which are NAE intermediates) may make *Elovl4b*^*−/−*^ zebrafish more susceptible to inflammation.

In contrast, as a possible compensatory measure, the levels of C26 VLC-PUFA containing acylcarnitine (ACar 26:6) increased in male *Elovl4b*^*−/−*^ fish compared with wild type, as well as PE 40:3 and 1,2-dimyristoyl-sn-glycero-3-phosphoethanolamine (DMPE) 16:0_20:3. PE 40:3 and DMPE 16:0_20:3 contain LC-PUFAs but have never been linked with ELOVL4 inactivity. Increased concentrations of the DMPE phospholipid headgroup have been linked to a lower conformational stability of rhodopsin (an important receptor for phototransduction) in dodecyltrimethylammonium bromide and octylglucoside detergent systems (24).

### *Elovl4b* ablation does not affect gross morphology but leads to the abnormal deposition of neutral lipids in the RPE

**Fig. 7:**
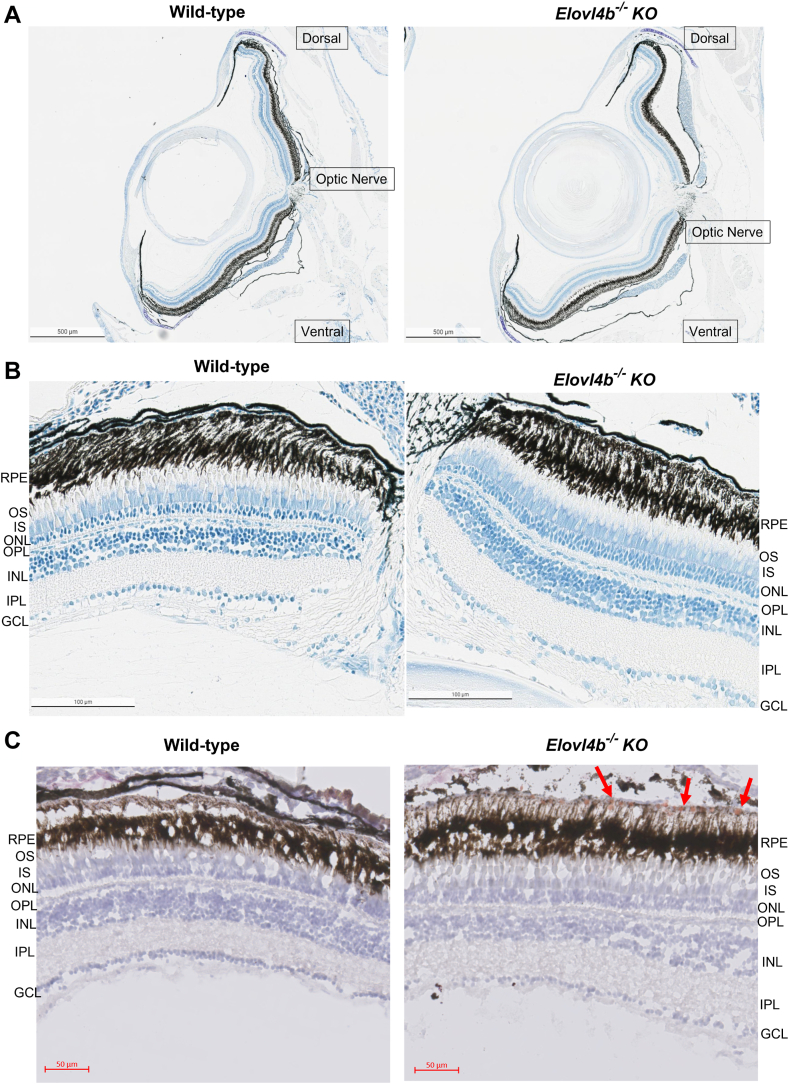
Gross histology and ORO stain. Toluidine blue-stained retinal sections of 1-year-old wild-type and homozygous *Elovl4b* KO fish. We observed no significant differences in the gross morphology and lamination of the retinas from both groups. Images were taken at 4× (A) and 40× (B) magnification. Cryosections of 1-year-old age-matched male wild-type and homozygous *Elovl4b* KO zebrafish retinas, treated with ORO to stain lipids (red) and counterstained with hematoxylin (blue) (C). The red arrows point to lipid-rich ORO-stained puncta localizing in the basal RPE of homozygous *Elovl4b* KO mutants. Images were taken at 40× with a Zeiss AxioScan slide scanner. GCL, retinal ganglion cell layer; INL, inner nuclear layer; IPL, inner plexiform layer; IS, inner segment; ONL, outer nuclear layer; OPL, outer plexiform layer; OS, outer segment; RPE, retinal pigment epithelium.

We found no difference in the gross morphology and lamination of the toluidine blue-stained retinas of adult homozygous *Elovl4b* KO fish compared with wild-type fish (Fig. 7A, B). However, we found ORO-stained lipid droplets situated in the basal RPE of the *Elovl4b*^*−/−*^ mutant fish that were not present in wild-type adults (Fig. 7C).

### *Elovl4b* ablation disrupts visual behavior in larval zebrafish

**Fig. 8:**
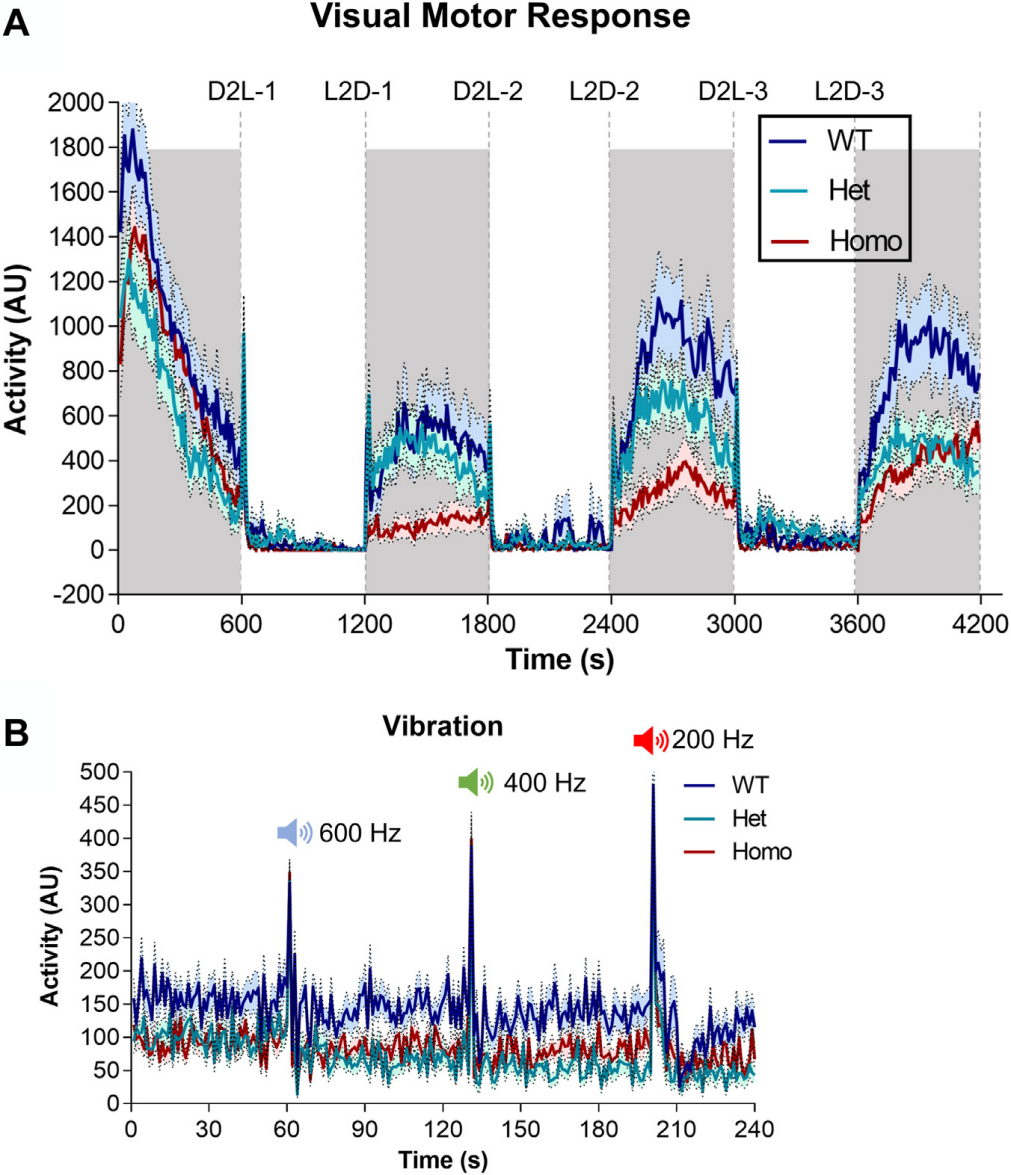
*Elovl4b* KO larvae show a diminished visual-motor response. A: Activity plot of zebrafish larvae response to light stimulus in alternating light-dark cycles. Homozygous and heterozygous *Elovl4b* KO fish show reduced activity in response to light-dark cycles compared with WT controls. B: Activity plot of control experiments showing no significant difference in larval response to different sound frequencies. Shaded region = dark. Data plotted as mean ± SEM, *N* = 32. AU, arbitrary unit; D2L, dark to light transition; Het, heterozygous *Elovl4b* KO mutant; Homo, homozygous *Elovl4b* KO mutant zebrafish larvae; L2D, light to dark transition.

To assess visual behavioral function in *Elovl4b* deletion mutant fish, we performed VMR experiments at 5 days post-fertilization (dpf). The VMR assay measures the larval zebrafish’s activity level in response to light stimulus. It is a well-characterized larval response showing a spike in activity in response to light onset (VMR-ON) and light offset (VMR-OFF) with a return to baseline activity levels (25, 26). We observed a marked decrease in activity levels of the *Elovl4*^*−/−*^ larvae in the light-to-dark phases compared with heterozygous mutants and wild-type controls (Fig. 8A). This reduction in activity is most clearly observed under dark conditions. As a control, we tested their response to different frequency sounds (Fig. 8B) with the same set of fish and found no significant difference among the groups.

We investigated the VMR-ON and VMR-OFF responses of 6 dpf larvae in a separate experiment and found that the activity of each group peaked within 2–3 s after light-off but returned to baseline within 10–15 s after peak activity (Supplemental Fig. S2A,B). However, *Elovl4*^*−/−*^ larvae had a muted response in the VMR-OFF test (Supplemental Fig. S2B). This result shows that *Elovl4b* ablation distinctly affects the VMR-OFF response in 6 dpf zebrafish larvae.

## Discussion

Understanding the implications of retinal VLC-PUFA depletion in the absence of confounding factors induced by protein mislocalization is critical for defining the roles of ELOVL4 and VLC-PUFAs in maintaining retinal health. This study provides the first in vivo animal model of targeted, complete VLC-PUFA depletion, where we demonstrate a direct link between *Elovl4b* loss, lipid profile changes, VLC-PUFA depletion, and poor visual outcomes.

We used CRISPR-Cas9 to generate a 56-bp deletion mutation in exon 2 of zebrafish *Elovl4b*. Fortunately, despite the expression of *Elovl4b* in critical organs like the gonads and pineal gland (10), homozygous *Elovl4b* KO mutant fish are fertile and appear normal. This is likely because of zebrafish’s duplicate genome in which two copies of *Elovl4* exist with differing expression patterns and functions. Although *Elovl4b′s* gene duplicate, *Elovl4a* cannot synthesize VLC-PUFAs, it can make VLC-SFAs to ensure normal function in diverse extraocular tissues, especially the nervous system, the integument, and possibly the reproductive organs. The divergent function and tissue distribution of the *Elovl4* gene duplicates is not unique in zebrafish. For example, zebrafish transducin is encoded by the duplicate genes *Gnb1a* and *Gnb1b*, which are also reported to have differing functions and expression patterns in the retina (27).

The deletion mutation caused a corresponding reduction in mRNA levels of *Elovl4b* in brain, retina, and RPE homogenates of *Elovl4b*^*+/−*^ and *Elovl4b*^*−/−*^ fish. The low levels of RT-PCR product from *Elovl4b* in the wild-type brain were likely because of Elov4b′s expression in the pineal gland, whereas the *Elovl4b* RT-PCR signal in the RPE may be attributed to endogenous expression of *Elovl4b* in RPE cells.

The near complete loss of C30-C36 VLC-PUFAs in homozygous *Elovl4b* mutant zebrafish was accompanied by an accumulation of C24-28 VLC-PUFAs in lipid extracts from their whole eyes (Fig. 4A–C). It was previously established that when heterologously expressed in *Saccharomyces cerevisiae*, *Elovl4b* synthesizes C26 to C36 n-3 and n-6 VLC-PUFAs (Fig. 1) (10). While this may be the case, we still found C26 and C28 n-3 and n-6 VLC-PUFAs in the eyes of *Elovl4b* KO fish. Likely, other ELOVLs (including perhaps *Elovl4b′s* gene duplicate, *Elovl4a*) can elongate to C28 but no further, or the trace amounts of C28 VLC-PUFAs in their feed may have been incorporated into eye tissue (supplemental Fig. S3). Serrano *et al.* (28) determined that the levels of LC-PUFAs in feed formulations and the mode of rearing can impact the levels of VLC-PUFAs in the eyes and other tissues of gilthead sea bream. Although the biosynthetic pathways of certain VLC-PUFAs may be redundant in zebrafish, this study clarifies that loss of VLC-PUFA >C28 is associated with a retinal and functional phenotype. This suggests that various strategies to increase retinal levels of these VLC-PUFAs through oral supplementation, gene augmentation, and other methods could have beneficial retinal health effects.

Our lipidomics data revealed differences in the lipid profiles of male and female wild-type and *Elovl4b*^*−/−*^ zebrafish eyes. There were significant reductions in VLC-PUFA-containing phospholipids in male and female *Elovl4b*^*−/−*^ zebrafish eyes as well as the substantial changes in a few additional lipid groups in the male *Elovl4b*^*−/−*^ zebrafish (Fig. 5). The relative absence of pleiotropy in the altered lipid species (≤10 in total) between the *Elovl4b* KO and wild-type groups suggests a narrowed function of *Elovl4b* in fish and the specificity of the gene-deletion induction strategy. We likewise detected sex-specific differences in the regulation of some lipid classes between the wild-type and *Elovl4b*^*−/−*^ mutant groups. For instance, more lipid groups, like lysolipids and VLC-PUFA-containing PCs, were significantly downregulated in *Elovl4b*^*−/−*^ mutant male tissue but not in their female counterparts (Fig. 6). In addition, there was a significant increase in the levels of a VLC-PUFA-containing ACar 26:6 in male *Elovl4b*^*−/−*^ fish eyes compared with wild type (Fig. 5). Increased levels of ACars are associated with FA oxidation disorders such as diabetic retinopathy (29). Differences in ocular lipid profile changes between male and female fish may influence the manifestation and severity of *Elovl4b* deficiency in these fish, but the mechanisms underlying these intriguing sex differences and their physiological significance remain to be explored.

We further evaluated the lipidomics data using the lipid ontology (LiON) database, which can rank and group enriched lipids in group comparisons according to various functional, cellular component, physical or chemical property, and lipid classification subcategories (30). The LiON enrichment analysis showed that glycerolipids, lipids with neutral head groups, lipids associated with lipid storage, droplet formation, and FAs <18 carbons in chain length, amongst others, were enriched in male and female *Elovl4b*^*−/−*^ fish eyes compared with sex-matched controls (supplemental Fig. S4). Moreover, some lipids with intrinsic negative curvatures that are upregulated in *Elovl4b* KO eyes (e.g., ceramides, PEs, and diacylglycerols) might have implications in photoreceptor membrane stability and membrane dynamics where the photoreceptor disks and lamellae rely on distinct curvature patterns. We performed lipid analyses on whole eyes because of the miniature size of zebrafish eyes—which need to be pooled for sufficient lipid extraction and detection. In the future, it would be interesting to evaluate retinal- and RPE-specific changes in the lipid profile of these groups.

*Elovl4b*^*−/−*^ fish displayed normal gross morphology and lamination in corroboration with previous studies by Dasyani *et al.* (31) on larval *Elovl2* knockdown crispants. Given that zebrafish regenerate neurons, it is not remiss to speculate that this intrinsic ability would mask any evidence of retinal degeneration. However, our *Elovl4b*^*−/−*^ fish had evidence of lipid accumulation within the basal RPE layer, a morphological correlate with the changes in C24 to C28 VLC-PUFAs and shorter-chain FAs observed in our GC-MS and lipid ontology studies (Fig. 4 and supplemental Fig. S4). The lipid abnormalities observed in the RPE of *Elovl4b* KO zebrafish are consistent with research on retinal degenerations in humans (32) and *Rp1l1* mutant zebrafish (20). Moreover, incubating fetal bovine RPE cells with high concentrations of phytanic acid (a shorter chain branched FA) elicited the formation of abnormal lipid-containing vacuoles (33)—which suggests that high levels of shorter-chain FAs can contribute to pathogenic lipid deposits in RPE cells. Nevertheless, further investigation is needed to evaluate other abnormalities like photoreceptor swelling, abnormal cell proliferation, vascular endothelial growth factor upregulation, changes in metabolism, signaling, energetics, or any other ultrastructural changes of the retinal and RPE cells, which may be deleterious to the *Elovl4b* KO fish.

Our VMR studies revealed that *Elovl4b*^*−/−*^ larvae had significantly reduced activity levels in response to the light-dark cycles compared with wild-type controls, whereas heterozygous *Elovl4b* KO larvae had an intermediate activity level response (Fig. 8A). By the fourth iteration of the dark-to-light cycle, the homozygous and heterozygous fish had similar activity levels in the dark, possibly indicating an adaptation to the light stimulation cycles (Fig. 8A). In agreement with Dasyani *et al.* (31), we observed significant changes in the VMR-OFF response of our 6 dpf larvae (supplemental Fig. S2B).

ELOVL4 dysfunction is involved in other disorders ranging from ichthyosis to spinocerebellar ataxia. We did not fully assess these fish’s motor or neurological function, but our *Elovl4b* heterozygous and homozygous mutant fish look and behave otherwise normally. VMR-ON responses are thought to reflect cone function, whereas VMR-OFF responses are associated with rod responses (31, 34). However, more time-course experiments, possibly involving electroretinograms and optokinetic and optomotor response tests—throughout the life span of the fish—from larvae to adulthood can be used to determine if Elovl4 dysfunction results in primarily a rod or cone dystrophy, or both.

Our studies demonstrate that *Elovl4* haploinsufficiency leads to decreased levels of ocular VLC-PUFAs and altered VMR responses in heterozygous *Elovl4b* KO fish. This finding has significant implications for AMD and STGD3 disease, where ocular VLC-PUFA depletion is incomplete. Although clinical studies have shown that diet can affect the severity of STGD3 disease (35), supplementing a regular diet with fish oil did little to improve the visual outcomes of patients with STGD3 in a cohort study (36). Conversely, wild-type and *Elovl4* conditional-KO mice gavage-fed C32:6n-3 VLC-PUFA had improved visual acuity and electroretinogram measurements from baseline (14). Perhaps a more direct approach with specific VLC-PUFAs, administered in adequate concentrations, would yield positive patient results.

In conclusion, we have successfully generated homozygous *Elovl4b* KO zebrafish using CRISPR-Cas9. We found that *Elovl4b* ablation causes VLC-PUFA depletion and significant alterations in the ocular lipid profiles of homozygous *Elovl4b* mutant zebrafish, whereas haploinsufficient heterozygous mutant fish present an intermediate phenotype. In addition, we demonstrated that the global loss of *Elovl4b* and consequent loss of VLC-PUFAs >C28 affect larval visual behavior significantly and generate subtle retinal morphological changes, including oil droplets in the RPE. This exciting finding sheds new light on the biosynthetic pathway of VLC-PUFAs in zebrafish and elucidates the effect of C30-C36 VLC-PUFA depletion on ocular function and retinal morphology. This zebrafish model is the first long-lived in vivo animal model of global VLC-PUFA depletion, and the data generated from these models would be useful in developing future therapies against VLC-PUFA-mediated pathologies. In the future, it will be interesting to compare these VLC-PUFA-depleted fish with fish having known STGD3 mutations and to attempt rescue with dietary VLC-PUFA supplements.

## Data availability

The data supporting this study’s findings are referenced or contained within the article.

## Supplemental data

This article contains supplemental data 37, 38, 39, 40, 41, 42, 43, 44, 45, 46.

## Conflict of interest

The authors declare that they have no conflicts of interest with the contents of this article.
