# Supplementary material for: Elovl4b knockout zebrafish as a model for ocular very-long-chain PUFA deficiency
Source: J Lipid Res. 2024 Feb 10;65(3):100518. doi: 10.1016/j.jlr.2024.100518 (PMC10940177; doi:10.1016/j.jlr.2024.100518)
Supplement: Supplemental data [file mmc1.pdf]

SUPPORTING INFORMATION

Elovl4b Knockout Zebrafish as a Model for Ocular Very-Long-Chain Polyunsaturated Fatty Acid Deficiency

Uzoamaka Nwagbo [1,2], Saba Parvez, PhD [1], J. Alan Maschek, PhD [3],

Paul S. Bernstein, MD, PhD [1,2 *]

[1]Department of Ophthalmology and Visual Sciences, John A. Moran Eye Center, University of Utah, Salt Lake City, UT 84132, USA

[2]Department of Pharmacology and Toxicology, University of Utah, Salt Lake City, UT 84112, USA

[3]Metabolomics Core, University of Utah Health Sciences Center, Salt Lake City, UT 84132, USA

Supplemental Materials and Methods

Supplemental Table S1

Supplemental Figures S1–4

Supplemental References

Supplemental Materials and Methods

Chemicals

The triacontanoic acid (C30:0) standard for gas chromatography-mass spectrometry analyses was purchased from Sigma-Aldrich (St. Louis, MO). Silica gel glass-encased solid-phase extraction cartridges (500 mg/6 mL) were purchased from Sorbent Technology (Norcross, GA). Bovine retina VLC-PUFAs were extracted and used as VLC-PUFA standards to establish identity and GC-MS retention times as described in prior work from our laboratory (37). For lipid analysis, LC-MS-grade solvents and mobile phase modifiers were obtained from Honeywell Burdick & Jackson (Morristown, NJ; acetonitrile, isopropanol, formic acid), Fisher Scientific (Waltham, MA; methyl tert-butyl ether) and Sigma–Aldrich/Fluka (St. Louis, MO; ammonium formate, ammonium acetate). We used EquiSPLASH® LIPIDOMIX Quantitative Mass Spec Internal Standard (Avanti Polar Lipids Cat. No. 330731), palmitoyl-L-carnitine-d3 (chloride) (Cayman Chemical Item No. 26569), and d31 palmitic acid (Cayman Chemical Item No. 16497) as internal standards.

GC-MS

Lipid extraction and purification of lipids with solid-phase extraction

We compared the levels of ocular VLC-PUFAs from five-month-old wild-type, heterozygous, and homozygous *Elovl4b* KO zebrafish. Fish were euthanized by ice-chilled water immersion. After death was established, microdissection was conducted, and two pairs of eyes were pooled for each sample. We extracted whole-eye lipids using published procedures from our laboratory (2). Briefly, each sample was homogenized in metal tubes using a BioSpec mini bead-beater and Fisher Scientific ice-bath sonicator. Total lipids were extracted and transferred using glass pipettes to clean glass vials using three 1 mL volumes of hexane/isopropanol (3:2 vol/vol), then dried under nitrogen gas. The film was resuspended in 200 μL of hexane and 2 mL of 4% HCl in MeOH, vortexed, and then placed in the incubator at 80 °C for 4 h for saponification to form fatty acid methyl esters (FAMEs). 2 mL hexane and 4 mL distilled water were added into the FAME mixture for liquid-liquid phase extraction. The mixture was vortexed and

centrifuged at ~1300 *g* for 5 min. Then, the upper (organic) layer was recovered using glass pipettes and transferred to a clean glass vial. An additional 2 mL of hexane was added to the reaction vial and vortexed and centrifuged for further recovery of the organic layer. This process was repeated three times. The final recovered organic layer was then washed with 3-4 volumes of distilled water until the pH of the solution was 7.0. This was done to remove underivatized fatty acids in the extract. The hexane layers were combined and dried under nitrogen gas. We performed solid-phase extraction (SPE) using silica gel glass-encased SPE cartridges with hexane–ether (4:1 vol/vol) to isolate and purify the FAME extracts. The FAME extracts were dried under a stream of nitrogen gas, resuspended in 20 µL of nonane, and 5 µL of the sample was injected into the GC-MS instrument for VLC-PUFA analyses.

Instrumentation, chromatographic conditions and data analysis

We used an Agilent GC-MS (Agilent Technologies, Santa Clara, CA) equipped with a 5977B mass spectrometer, a single quadrupole mass detector, an 8890 GC, and an autosampler. This system is operated with Xcalibur and Chemstation software. The chromatographic separation was performed with Restek Rxi-5MS coated 5% diphenyl/95% dimethyl polysiloxane capillary column (30 m × 0.25 mm internal diameter, 0.25 µm film thickness). The detection and quantitation of VLC-PUFAs were performed using previously published methods using electron impact (EI) selected ion monitoring (SIM) modes to detect and quantify C24 to C36 VLC-PUFAs with *m/z* ratios of 79, 108, and 150 (37). Peak areas were normalized in reference to the triacontanoic acid standard. Fatty acid levels in this study are expressed as normalized peak areas.

LC-MS/MS

Sample preparation

Five pairs of 9-month-old zebrafish eyes from each experimental group were pooled and weighed in 10 mL glass vials. We adapted the protocol used by Harkiewicz et al., 2012 (21) to extract retinal lipids. Briefly, the tissue samples were homogenized using a probe sonicator, and two volumes of 2 mL of ice-cold HPLC/LC-MS grade dichloromethane/methanol (2:1, v/v) containing a total of 13 µL of EquiSPLASH, 10 µL of 10 µg/mL palmitoyl-L-carnitine-d3 (chloride) (Cayman item No. 26569), and 10 µL of the d31 palmitic acid (Cayman Chem 16497) as internal standards. Then, liquid-liquid phase extraction was performed by adding high-purity distilled water, vortexing the sample, and centrifuging at ~1300 *g* for 5 min. The lower phase was stored at -80 °C until resuspension in 250 µL of HPLC buffer solution (IPA/ACN/water) and analysis by the University of Utah's Metabolomics Core facility.

Mass spectrometry analysis of samples

Lipid extracts were separated on an Acquity UPLC CSH C18 column (2.1 x 100 mm; 1.7 µm) coupled to an Acquity UPLC CSH C18 VanGuard precolumn (5 × 2.1 mm; 1.7 µm) (Waters, Milford, MA) maintained at 65 °C connected to an Agilent HiP 1290 Sampler, Agilent 1290 Infinity pump, and Agilent 6545 Accurate Mass Q-TOF dual AJS-ESI mass spectrometer (Agilent Technologies, Santa Clara, CA). Samples were analyzed in a randomized order in both positive and negative ionization modes in separate experiments acquiring with the scan range *m/z* 100 – 1700. For positive mode, the source gas temperature was set to 225°C, with a drying gas flow of 11 L/min, nebulizer pressure of 40 psig, sheath gas temp of 350 °C and sheath gas flow of 11 L/min. VCap voltage was set at 3500 V, nozzle voltage at 500 V, fragmentor at 110 V, skimmer at 85 V, and octopole RF peak at 750 V. For negative mode, the source gas temperature was set to 300°C, with a drying gas flow of 11 L/min, a nebulizer pressure of 30 psig, sheath gas temp of 350°C and sheath gas flow 11 L/min. VCap voltage was set at 3500 V, nozzle voltage 75 V, fragmentor at 175 V, skimmer at 75 V and octopole RF peak at 750 V. Mobile phase A consisted of ACN:H_2O (60:40, *v/v*) in 10 mM ammonium formate and 0.1% formic acid, and mobile phase B consisted of IPA:ACN:H_2O (90:9:1, *v/v/v*) in 10 mM ammonium formate and 0.1% formic acid.

For negative mode analysis, the modifiers were changed to 10 mM ammonium acetate. The chromatography gradient for both positive and negative modes started at 15% mobile phase B and then increased to 30% B over 2.4 min. It then increased to 48% B from 2.4 – 3.0 min, then increased to 82% B from 3 – 13.2 min, then increased to 99% B from 13.2 – 13.8 min, where it was held until 16.7 min and then returned to the initial conditions and equilibrated for 5 min. Flow was 0.4 mL/min throughout, with injection volumes of 2 µL for positive and 8 µL for negative mode. Tandem mass spectrometry was conducted using iterative exclusion with the same LC gradient at collision energies of 20 V and 27.5 V in positive and negative modes, respectively.

Analysis of mass spectrometry data

For data processing, Agilent MassHunter (MH) Workstation and software packages MH Qualitiative and MH Quantitative were used. The pooled QC (n=8) and process blank (n=4) were injected throughout the sample queue to ensure the reliability of acquired lipidomics data. For lipid annotation, accurate mass and MS/MS matching was used with the Agilent Lipid Annotator library and LipidMatch (38). Results from the positive and negative ionization modes from Lipid Annotator were merged based on the class of lipid identified. Data exported from MH Quantitative were evaluated using Excel where initial lipid targets are parsed based on the following criteria. Only lipids with relative standard deviations (RSD) less than 30% in QC samples are used for data analysis. Additionally, only lipids with background AUC counts in process blanks that were less than 30% of QC were used for data analysis. In total, 926 lipids were in the final data table for statistical analysis. The parsed Excel data tables were normalized based on the ratio to class-specific internal standards and then to tissue mass prior to statistical analysis.

Statistical analysis and data visualization

Multivariate analysis was performed using MetaboAnalyst (39). Statistical models were created for the normalized data after logarithmic transformation (base 10) and Pareto scaling. Initial pass for the volcano plot used a fold change (FC) cut off of 1.5, with an adjusted p-value cut off of 0.05.

Visual motor response and vibration test

Thirty-two wild-type, heterozygous, and homozygous *Elovl4b* KO 5 days-post-fertilization (dpf) zebrafish larvae were placed in individual wells in flat bottom clear 96-well plates (Whatman Cat. No. 7701-1651 New Jersey, USA) with 200 µL of E3 medium. All behavioral assays were performed using the ZebraBox® system (ViewPoint Life Sciences, Lyon, France). This system provides a closed environment where larvae are not exposed to environmental ambient light and temperature fluctuations. Larval activity in each assay was recorded by ZebraLab® software (ViewPoint Life Sciences, Lyon, France) using a camera capturing at 30 frames per second and running in the quantization mode. The light and dark cycles were adapted from previously published protocols (40). Briefly, larvae were acclimatized to their new surroundings before the experiment**.** Light and dark cycles lasted 10 min each. The test consisted of four cycles of light on/off. In each light cycle, the fish were exposed to a white light stimulus at 100% intensity for 10 min followed by a 10 min dark period. The control experiment involved measuring the startle response to 20 ms of 200 Hz, 300 Hz, and 600 Hz acoustic vibration stimuli in dark conditions. The activity data (total movement/s) were exported from the software and analyzed using Microsoft Excel and GraphPad Prism.

In a separate experiment, we characterized the VMR-ON and VMR-OFF response through quantitating larval activity 60 sec before and after light on/off induction. The larvae were first dark acclimatized (0 - 300 s), then stimulated with white light (300 s – 600 s). The activity data (total movement/s) were exported from the software and analyzed using Microsoft Excel and GraphPad Prism.

Zebrafish Elovl4 is conserved between zebrafish and mammalian homologs

The *ELOVL4* gene is conserved in the vertebrate retina (41). Zebrafish putative *Elovl4* elongase genes are located on different chromosomes of the zebrafish genome. *Elovl4a* (gb|NM_200796|) is on chromosome 16, and *Elovl4b* (gb|NM_199972|) is on chromosome 23 (10). BLASTn analysis showed that the zebrafish *Elovl4a* and *Elovl4b* amino acid sequences shared similarity with human *ELOVL4*, with 100% query coverage and 68.31% and 67.94% identities, respectively. Sequence similarities to human *ELOVL4* were 83% for *Elovl4a* and 82% for *Elovl4b*. We performed amino acid sequence alignment using Clustal Omega Sequence Alignment Tool (42). Sequence alignment showed relative conservation in transmembrane domains, dioxy iron-binding HXXHH redox center motif, and carboxy-terminal dilysine signal (KXKXX) responsible for retention in the endoplasmic reticulum (**Supplemental Fig. S1**). However, the SOSUI transmembrane prediction algorithm (https://harrier.nagahama-i-bio.ac.jp/sosui/) shows Elovl4b has a more similar structure to human ELOVL4 (13, 43) than Elovl4a, as it gives five putative transmembrane domains for Elovl4b and human ELOVL4, but only four putative transmembrane domains for Elovl4a (44). The uniqueness of zebrafish Elovl4a from mammalian orthologues has been highlighted through substrate specificity and tissue expression pattern analyses by Monroig et al (10). However, this is the first time putative structural differences of zebrafish Elovl4a to Elovl4b and human ELOVL4 has been suggested.

A multiple sequence alignment of ELOVL4 proteins from different species was generated using Clustal Omega. Accession numbers of the protein sequences used for sequence comparison are as follows: human, NP_073563.1 (RefSeq); mouse, NP_683743.2 (RefSeq); *Danio rerio Elovl4a*, NP_957090.1 (RefSeq); *Danio rerio Elovl4b*, NP_956266.1 (RefSeq). The major difference in sequence between mammalian ELOVL4 and zebrafish Elovl4a and Elovl4b is at the N-terminal. Zebrafish Elovl4 protein sequences are also highly dissimilar at the C-terminal. Moreover, the carboxy-terminal dilysine signal (KXKXX) responsible for retention in the endoplasmic reticulum shows differences among the groups, with Elovl4b having two amino acids changed but still maintaining the KXKXX motif, while Elovl4a also has two amino acids changed and does not maintain the KXKXX motif (**Supplemental Fig.**

S1). According to the phylogenetic tree illustrated by Monroig et al. (2010), zebrafish Elovl4a clusters more closely with *T. rubripes* Elovl4, than zebrafish Elovl4b, which clusters more closely with the pufferfish *T. nigroviridis* orthologue (10).

Target gene	Primer	Primer sequence 5' to 3'	T_m (ºC)	Primer position	Fragment size	Accession No.	Reference
Elovl4b	Elovl4bF	5'-CACGCGCTCGTAAGGATAAT-3'	63.1	Exon 1-5	507 bp	NM_199972	Monroig *et al*. (2010) (10)
	Elovl4bR	5'-GGATGAACATTGTGCAGTGG-3'	62.5				
Elfα	ElfαF	5'-TACAAATGCGGTGGAATCGAC-3'	63.9	Exon 2-3	246 bp	NM_131263.1	Lang *et al*. (2015) (45)
	ElfαR	5'-GTCAGCCTGAGAAGTACCAGT-3'	64.3				

Supplemental Table S1. Sets of primers used for cDNA synthesis and RT-PCR

```
CLUSTAL O(1.2.4) multiple sequence alignment

Human_ELOVL4              MGLLDSEPGSVLNVVSTALNDTVEFYRWTWSIADKRVENWPLMQSPWPTLSISTLYLLFV     60
Mouse_Elovl4              MGLLDSEPGSVLNAMSTAFNDTVEFYRWTWTIADKRVADWPLMQSPWPTISISTLYLLFV     60
Zebrafish_Elovl4a         -----------MEIIQHIINDTVHFYKWSLTIADKRVEKWPLMDSPLPTLAISSSYLLFL     49
Zebrafish_Elovl4b         -----------METVVHLMNDSVEFYKWSLTIADKRVEKWPMMSSPLPTLGISVLYLLFL     49
                                     :: :   :**:*.**:*: :****** .**:*.** **:.**  ****:

Human_ELOVL4              WLGPKWMKDREPFQMRLVLIIYNFGMVLLNLFIFRELFMGSYNAGYSYICQSVDYSNNVH     120
Mouse_Elovl4              WLGPKWMKDREPFQMRLVLIIYNFGMVLLNLFIFRELFMGSYNAGYSYICQSVDYSNDVN     120
Zebrafish_Elovl4a         WLGPKYMQGREPFQLRKTLIIYNFSMVILNFFIFKELFLAARAANYSYICQPVDYSDDPN     109
Zebrafish_Elovl4b         WAGPLYMQNREPFQLRKTLIVYNFSMVLLNFYICKELLLGSRAAGYSYLCQPVNYSNDVN     109
                          * ** :*:.*****:* .**:***.**:**::* :**::.:  *.***:** *:**:: :

Human_ELOVL4              EVRIAAALWWYFVSKGVEYLDTVFFILRKKNNQVSFLHVYHHCTMFTLWWIGIKWVAGGQ     180
Mouse_Elovl4              EVRIAGALWWYFVSKGVEYLDTVFFILRKKNNQVSFLHVYHHCTMFTLWWIGIKWVAGGQ     180
Zebrafish_Elovl4a         EVRVAAALWWYFISKGVEYLDTVFFILRKKFNQISFLHVYHHCTMFTLWWIGIKWVAGGQ     169
Zebrafish_Elovl4b         EVRIASALWWYYISKGVEFLDTVFFIMRKKFNQVSFLHVYHHCTMFILWWIGIKWVPGGQ     169
                          ***:*.*****::*****:*******:*** **:************ ********* ***

Human_ELOVL4              AFFGAQLNSFIHVIMYSYYGLTAFGPWIQKYLWWKRYLTMLQLIQFHVTIGHTALSLYTD     240
Mouse_Elovl4              AFFGAQMNSFIHVIMYSYYGLTAFGPWIQKYLWWKRYLTMLQLVQFHVTIGHTALSLYTD     240
Zebrafish_Elovl4a         SFFGAHMNAAIHVLMYLYYGLAAFGPKIQKFLWWKKYLTIIQMVQFHVTIGHTALSLYSD     229
Zebrafish_Elovl4b         SFFGATINSGIHVLMYGYYGLAAFGPKIQKYLWWKKYLTIIQMIQFHVTIGHAAHSLYTG     229
                          :**** :*: ***:** ****:**** ***:****:***::*::********:* ***:.

Human_ELOVL4              CPFPKWMHWALIAYAISFIFLFLNFYIRTYKEPKKPKAGKTAMNGISA------NGVSKS     294
Mouse_Elovl4              CPFPKWMHWALIAYAISFIFLFLNFYTRTYNEPKQSKTGKTATNGISS------NGVNKS     294
Zebrafish_Elovl4a         CPFPKWMHWCLIGYALTFIILFGNFYYQTYRRQPRRDKPRALHNGASNGALTSSNGNTAK     289
Zebrafish_Elovl4b         CPFPAWMQWALIGYAVTFIILFANFYYQTYRRQPRLKTAKSAVNGVSM----STNGTSKT     285
                          **** **:*.**.**::**:** *** :**..  : .  ::  ** *       ** . .

Human_ELOVL4              EKQLMIENGKKQKNGKAKGD 314
Mouse_Elovl4              EKA--LENGKPQKNGKPKGE 312
Zebrafish_Elovl4a         LEEKPAESGRRRRKGRAKRD 309
Zebrafish_Elovl4b         --AEVTENGKKQKKGKGKHD 303
                                *.*: :::*: * :
```

Supplemental Figure S1. ELOVL4 sequence alignment. Sequence alignment of ELOVL4 shows conservation in different species. * represents identical amino acids; a colon (:) represents highly similar amino acids; a period (.) represents amino acids with slight similarities. The conserved dioxy iron-binding HXXHH redox center motif (red) and carboxy-terminal dilysine signal (KXKXX) responsible for retention in the endoplasmic reticulum (blue). The four (I-IV) conserved Elovl motifs are highlighted in yellow: I (KXXEXXDT), II (QXXFLHXXHH), III (NXXXHXXMYXYY), and IV (TXXQXXQ). Amino acid residues that differ from the human sequence in the highlighted areas are underlined. The second Elovl motif contains the HXXHH dioxy iron-binding motif. Image adapted from Betancor *et al.* (2020) (46).

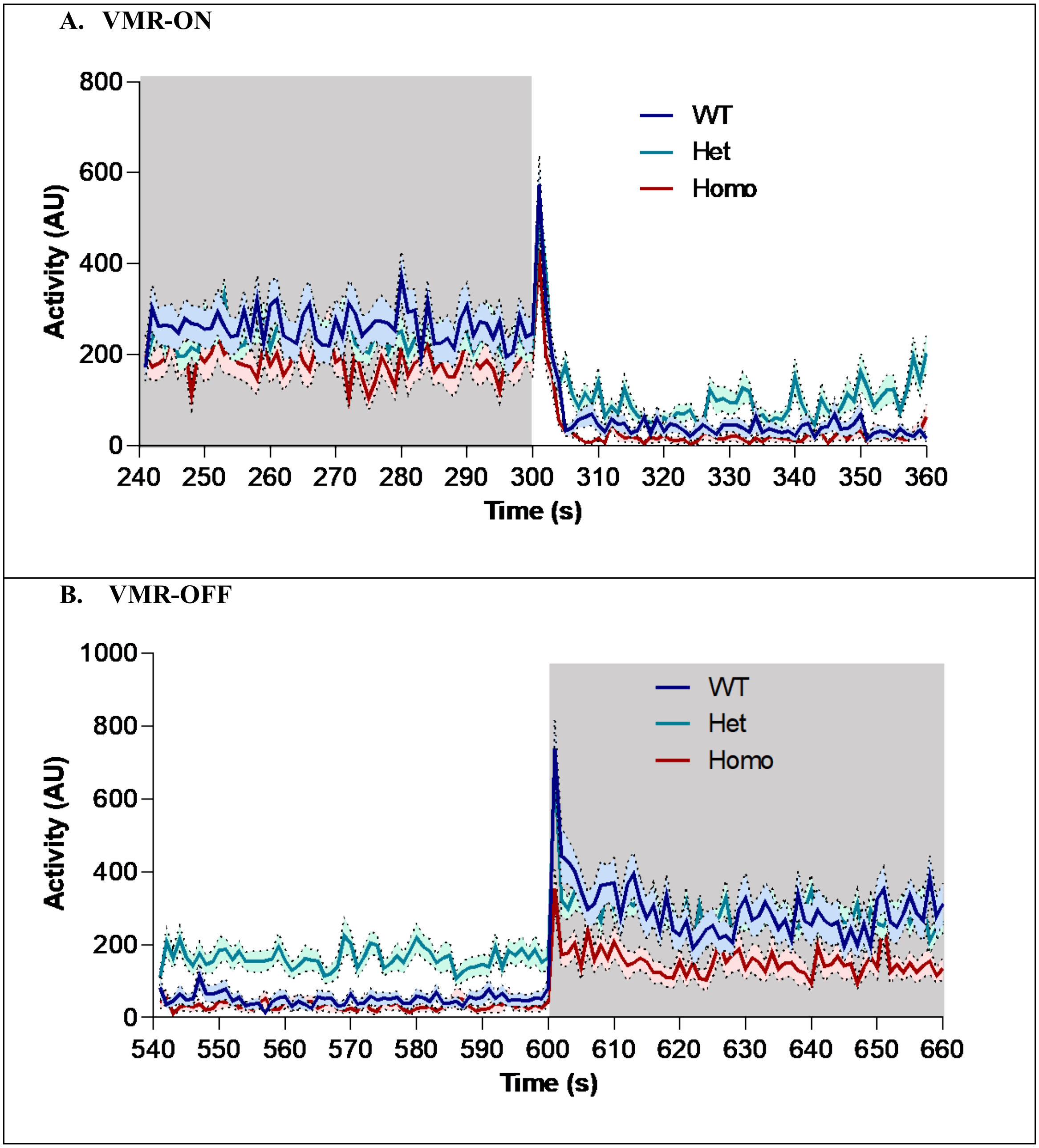

Supplemental Figure S2. VMR-ON and VMR-OFF analyses. A) VMR-ON response plot. **B)** VMR-OFF response plot. Data was recorded 60 s before and after a 100 ms white light stimulus. Shaded region= dark. Data plotted as mean +/- SEM, N= 32. AU - arbitrary unit. WT: wild-type; Het: heterozygous *Elovl4b* mutants; Homo: homozygous *Elovl4b* mutant zebrafish larvae.

A.

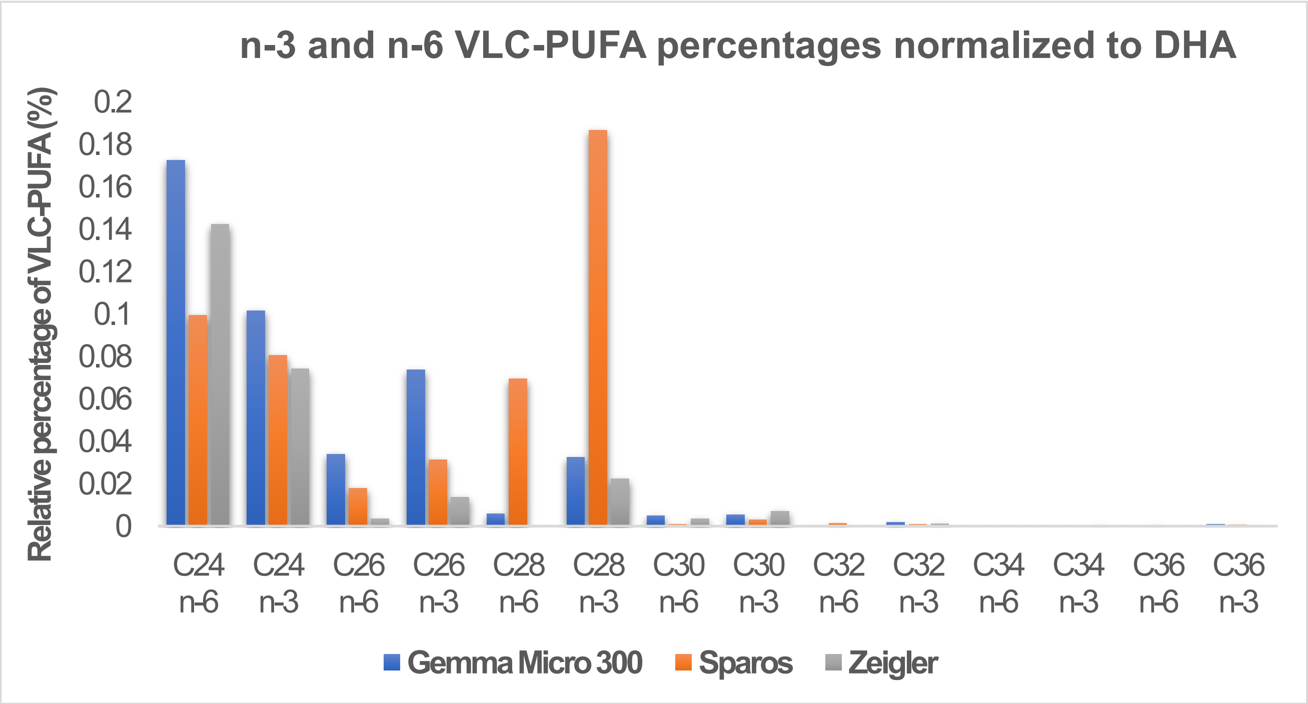

B.

Fish Feed	C18:1 n-9	C20:5 n-3 (EPA)	C22:6 n-3 (DHA)
**Gemma Micro 300**	34.6 %	15.5 %	29.9 %
**Sparos**	51.5 %	12.7 %	16.4 %
**Zeigler**	13.4 %	26.5 %	21.9 %

Supplemental Figure S3. Very-long chain fatty acid profiles of three commercial zebrafish feeds. **Graph A**. shows the percentages of n-3 and n-6 VLC-PUFAs in Gemma Micro 300 (blue), Sparos (orange), and Zeigler (gray) commercial adult zebrafish feeds normalized to the percentage of DHA. **Table B.** shows the percentages of the three major fatty acids in the commercial feeds. Short to long chain fatty acid analysis was performed using methods adapted from Gorusupudi et al. (14) Fatty acids were extracted from 100 mg of feed, and relative percentages were determined from the total fatty acids referenced in the C4-C24 FAME Mix (Supelco Cat. No. 18919-1AMP). N=1.

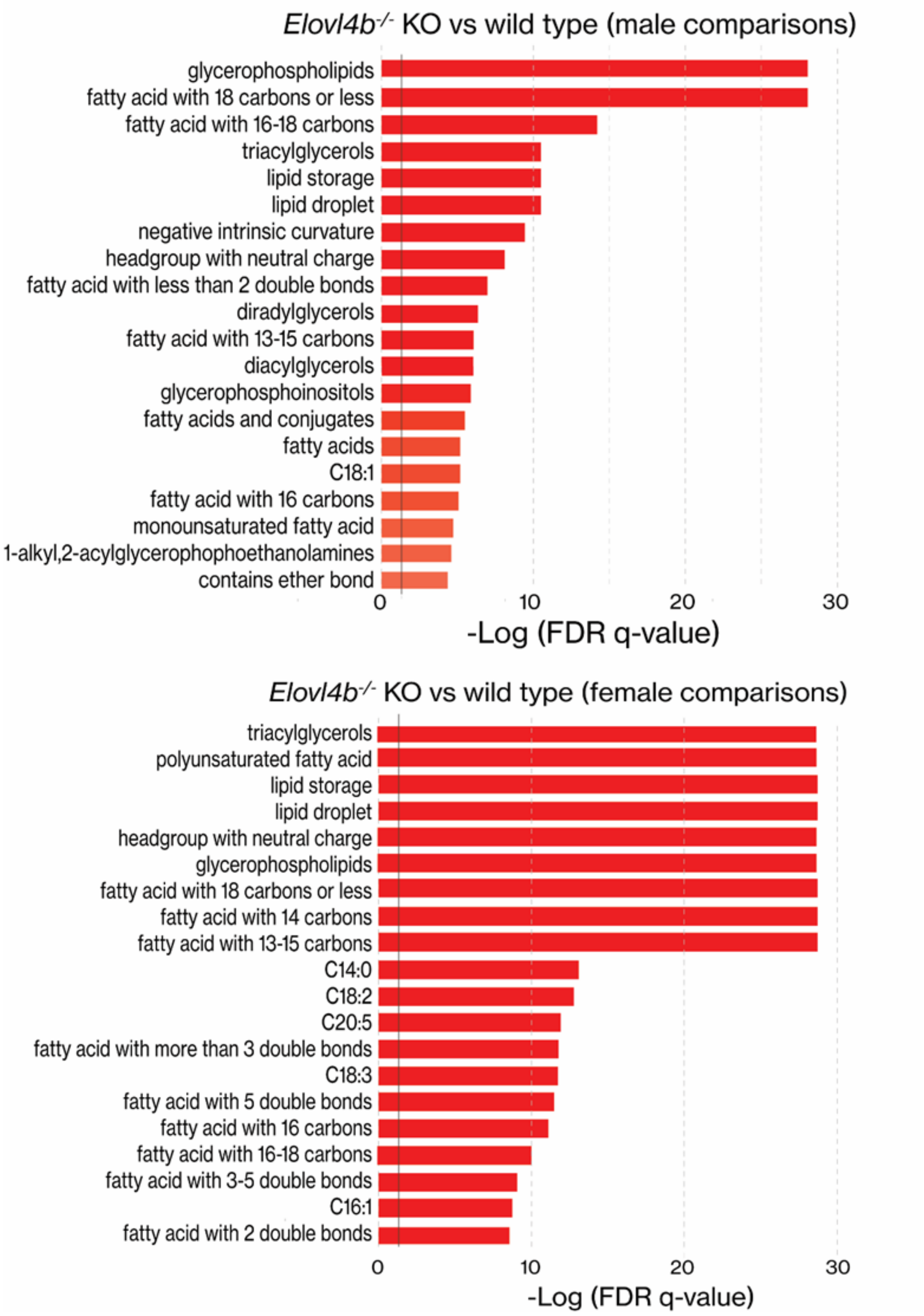

Supplemental Figure S4. Lipid ontology enrichment graphs. Enrichment graphs of the top 20 enriched lipid classifications of the lipids extracted from whole eyes of male and female *Elovl4b* KO fish compared to age- and sex-matched wild-type fish using a one-tailed t-test and the lipid ontology (LiON) enrichment analysis in ranking mode. *Elovl4b* KO vs. wild-type male (top) and *Elovl4b* KO vs. wild-type female (bottom) lipid enrichment comparisons. FDR= false discovery rate.

Supplemental References

2. Liu A, Chang J, Lin Y, Shen Z, Bernstein PS. Long-chain and very long-chain polyunsaturated fatty acids in ocular aging and age-related macular degeneration. Journal of Lipid Research. 2010;51(11).

10. Monroig O, Rotllant J, Cerdá-Reverter JM, Dick JR, Figueras A, Tocher DR. Expression and role of Elovl4 elongases in biosynthesis of very long-chain fatty acids during zebrafish Danio rerio early embryonic development. Biochim Biophys Acta. 2010;1801(10):1145-54. Epub 2010/07/06. doi: 10.1016/j.bbalip.2010.06.005. PubMed PMID: 20601113.

13. Hopiavuori BR, Anderson RE, Agbaga MP. ELOVL4: Very long-chain fatty acids serve an eclectic role in mammalian health and function. Prog Retin Eye Res. 2019;69:137-58. Epub 2019/04/16. doi: 10.1016/j.preteyeres.2018.10.004. PubMed PMID: 30982505; PubMed Central PMCID: PMC6688602.

14. Gorusupudi A, Rallabandi R, Li B, Arunkumar R, Blount JD, Rognon GT, et al. Retinal bioavailability and functional effects of a synthetic very-long-chain polyunsaturated fatty acid in mice. Proc Natl Acad Sci U S A. 2021;118(6). Epub 2021/02/03. doi: 10.1073/pnas.2017739118. PubMed PMID: 33526677; PubMed Central PMCID: PMC8017942.

21. Harkewicz R, Du H, Tong Z, Alkuraya H, Bedell M, Sun W, et al. Essential Role of ELOVL4 Protein in Very Long Chain Fatty Acid Synthesis and Retinal Function. Journal of Biological Chemistry. 2012;287(14):11469-80. doi: 10.1074/jbc.m111.256073.

37. Liu A, Terry R, Lin Y, Nelson K, Bernstein PS. Comprehensive and sensitive quantification of long-chain and very long-chain polyunsaturated fatty acids in small samples of human and mouse retina. Journal of Chromatography A. 2013;1307:191-200. doi: 10.1016/j.chroma.2013.07.103.

38. Koelmel JP, Kroeger NM, Ulmer CZ, Bowden JA, Patterson RE, Cochran JA, et al. LipidMatch: an automated workflow for rule-based lipid identification using untargeted high-resolution tandem mass spectrometry data. BMC Bioinformatics. 2017;18(1):331. Epub 20170710. doi: 10.1186/s12859-017-1744-3. PubMed PMID: 28693421; PubMed Central PMCID: PMC5504796.

39. Xia J, Sinelnikov IV, Han B, Wishart DS. MetaboAnalyst 3.0--making metabolomics more meaningful. Nucleic Acids Res. 2015;43(W1):W251-7. Epub 20150420. doi: 10.1093/nar/gkv380. PubMed PMID: 25897128; PubMed Central PMCID: PMC4489235.

40. Messchaert M, Dona M, Broekman S, Peters TA, Corral-Serrano JC, Slijkerman RWN, et al. Eyes shut homolog is important for the maintenance of photoreceptor morphology and visual function in zebrafish. Plos One. 2018;13(7). doi: ARTN e0200789

10.1371/journal.pone.0200789. PubMed PMID: WOS:000440006600015.

41. Lagali PS, Liu J, Ambasudhan R, Kakuk LE, Bernstein SL, Seigel GM, et al. Evolutionarily conserved ELOVL4 gene expression in the vertebrate retina. Investigative Ophthalmology and Visual Science. 2003;44(7):2841-50. doi: 10.1167/iovs.02-0991.

42. Goujon M, McWilliam H, Li WZ, Valentin F, Squizzato S, Paern J, et al. A new bioinformatics analysis tools framework at EMBL-EBI. Nucleic Acids Research. 2010;38:W695-W9. doi: 10.1093/nar/gkq313. PubMed PMID: WOS:000284148900113.

43. Molday RS, Zhang K. Defective lipid transport and biosynthesis in recessive and dominant Stargardt macular degeneration. Prog Lipid Res. 2010;49(4):476-92. Epub 20100713. doi: 10.1016/j.plipres.2010.07.002. PubMed PMID: 20633576; PubMed Central PMCID: PMC2946192.

44. Hirokawa T, Boon-Chieng S, Mitaku S. SOSUI: classification and secondary structure prediction system for membrane proteins. Bioinformatics. 1998;14(4):378-9. doi: 10.1093/bioinformatics/14.4.378. PubMed PMID: 9632836.

45. Lang X, Wang L, Zhang Z. Stability evaluation of reference genes for real-time PCR in zebrafish (Danio rerio) exposed to cadmium chloride and subsequently infected by bacteria Aeromonas hydrophila. Aquat Toxicol. 2016;170:240-50. Epub 20151202. doi: 10.1016/j.aquatox.2015.11.029. PubMed PMID: 26675370.

46. Betancor MB, Oboh A, Ortega A, Mourente G, Navarro JC, de la Gandara F, et al. Molecular and functional characterisation of a putative elovl4 gene and its expression in response to dietary fatty acid profile in Atlantic bluefin tuna (Thunnus thynnus). Comparative Biochemistry and Physiology B-Biochemistry & Molecular Biology. 2020;240. doi: ARTN 110372

10.1016/j.cbpb.2019.110372. PubMed PMID: WOS:000508741700003.
